# Corrigendum

**DOI:** 10.1093/jxb/eru445

**Published:** 2014-12-12

**Physiological and molecular responses to drought in *Petunia*: the importance of stress severity**

Jongyun Kim, Anish Malladi, and Marc W. van Iersel

*Journal of Experimental Botany* (2012) **63** (18): 6335–6345. doi: 10.1093/jxb/ers285

In the above paper, Figure 1 and a subset of the data in Figure 2 were previously published in a proceedings paper of the Southern Nursery Association Research Conference (Kim J, Malladi A, van Iersel MW. 2011. Physiological responses of petunia to different levels of drought stress. Proceedings of Southern Nursery Association Research Conference **56**, 46–51).

